# Area-Level Correlates of Inpatient Care Self-Sufficiency in Subnational Health Planning Areas: A Disease-Stratified Ecological Study from Aichi Prefecture, Japan

**DOI:** 10.3390/healthcare14193322

**Published:** 2026-10-06

**Authors:** Wataru Sonta, Kikue Sato

**Affiliations:** 1 Medical Innovation Core for Society, Graduate School of Medicine, Kyoto Prefectural University of Medicine, Kyoto 602-0841, Japan; 2 Medical IT Center, Nagoya University Hospital, Nagoya 466-8560, Japan; sato.kikue.e4@f.mail.nagoya-u.ac.jp

**Keywords:** health services accessibility, regional health planning, small-area analysis, catchment area, health, hospitalization, health services needs and demand, cross-sectional studies, aged, Japan

## Abstract

**Background/Objectives:** Japan organizes inpatient care through secondary medical areas (SMAs), but evidence linking area-level care self-sufficiency with population need, healthcare supply, and socioeconomic context is limited. We quantified disease-stratified self-sufficiency across Aichi Prefecture and examined its ecological correlates. **Methods:** This cross-sectional ecological study analyzed fiscal-year 2024 Diagnosis Procedure Combination discharges from 203 of 276 acute-care hospitals across 11 SMAs. The dataset comprised 396 cells (11 SMAs × 18 Major Diagnostic Categories × surgical/non-surgical strata) linked to census, healthcare-resource, household, and pooled 2020–2024 standardized mortality ratio (SMR) data. Pre-specified single-predictor regressions at the SMA level (*n* = 11) were supplemented by spatial and cluster-robust sensitivity analyses. Self-sufficiency represented observed resident-to-provider flows within the participating hospital network. **Results:** Mean self-sufficiency ranged from 34.0% to 88.3% across SMAs (intra-class correlation, 0.54) and was 8.5 percentage points lower for surgical cells (95% CI, 5.9–11.1). Greater shares of residents aged ≥75 years (β = −12.80; 95% CI, −22.84 to −2.75) and households containing an elderly member (β = −13.53; 95% CI, −23.02 to −4.05) were the strongest negative correlates. Supply-density estimates were consistently positive; in the cell-level sensitivity model, physician, general-bed, and psychiatric-bed densities were associated with above-median self-sufficiency (ORs, 2.51–3.16). Exploratory analyses suggested differing patterns across acute and chronic conditions. **Conclusions:** Self-sufficiency varied substantially across planning areas and was associated with demographic, household, supply, and mortality characteristics. These findings support disease-specific evaluation of regional concentration versus local completion but do not establish causal or individual-level relationships. Multi-prefecture studies with individual-level case-mix adjustment are needed before resource-allocation applications.

## 1. Introduction

Global life expectancy reached 73.3 years in 2024, and the number of people aged 60 years or older is projected to rise from 1.1 billion in 2023 to 1.4 billion by 2030 [1,2]. This demographic transition is only partially matched by gains in healthy life expectancy, and good health in old age is unequally distributed both between and within societies [3,4]. As chronic-disease burden, multimorbidity, and the costs of long-term care escalate, the central challenge for health systems is no longer to expand service volume but to allocate limited resources so that appropriate care remains available where residents live [5,6]. Under the United Nations Decade of Healthy Ageing (2021–2030), health systems are expected to plan and integrate primary, acute, and long-term care within geographically defined administrative units [2,7].

Japan is an informative case for this debate. It has been a global leader in life expectancy for more than three decades, having achieved a comprehensive, equitable, and affordable national health-insurance system that provides near-universal access to care [8,9,10]. To preserve both equity and sustainability under intensifying demographic and fiscal stress, the Japanese government has, since 1985, organized inpatient-care planning through 335 secondary medical areas (SMAs; *niji-iryōken*), each expected to provide self-contained acute care for its resident population [11]. Reimbursement and case-mix accounting in acute hospitals across the country are standardized through the Diagnosis Procedure Combination (DPC) system, which classifies each inpatient episode into one of 18 Major Diagnostic Categories (MDCs) by primary disease and procedure type [12]. Whether this planning model can continue to deliver appropriate care in the world’s most aged population is relevant to other countries that organize universal health coverage through sub-national regions [5,9,13].

The large international literature documents substantial and persistent small-area variation in healthcare utilization and expenditure that cannot be fully explained by underlying population health needs [14,15,16], a sizeable share of delivered services is reportedly low-value or unnecessary [17,18], and the Organisation for Economic Co-operation and Development (OECD) has argued that integrated, regionally coordinated models of care are a key mechanism for sustainable system performance [19]. Within Japan, three lines of area-level evidence have accumulated. First, nationwide analyses have described the geographic distribution of healthcare resources and inter-regional patient flow, suggesting that patients in less-resourced areas tend to travel to better-equipped metropolitan hospitals [20]. Second, SMA-level ecological studies have separately characterized disparities in home-care utilization [21], the distributional structure of medical and long-term-care resources [22], sub-prefectural variation in healthcare expenditure [23], and the relationships between healthcare resources, mortality, and healthy life expectancy [24,25]. Third, area-level socioeconomic conditions have repeatedly been linked to cause-specific mortality and access to care [26,27,28]. However, three questions remain unanswered. No study has quantified inpatient care self-sufficiency (the proportion of residents’ admissions completed within their own SMA) across all 18 MDCs and surgical strata using acute-care discharge records covering every SMA of a prefecture. No study has linked this flow outcome simultaneously to supply density, cause-specific mortality need, and household-composition socioeconomic context within a single analytic dataset. Consequently, whether the disease-specific balance between regional concentration and local completion of inpatient care follows a systematic clinical pattern, and which area characteristics accompany it, remains unknown. Without such evidence, planners in Japan and in other aging societies that allocate hospital capacity through sub-national regions must rely on separate, unlinked indicators [5,19].

This study examined the area-level associations of healthcare supply, cause-specific population need, and household-level socioeconomic structure with the self-completion, outflow, and inflow of inpatient care within secondary medical areas of Japan. Using fiscal-year 2024 (FY2024) DPC inpatient records from 203 participating acute-care hospitals across the 11 SMAs of Aichi Prefecture, we applied a disease-stratified ecological design to describe area-level correlates of inpatient care self-sufficiency at the SMA level. To our knowledge, this is the first study to quantify disease-stratified inpatient care self-sufficiency across all SMAs of a Japanese prefecture and to examine its demographic, supply, need, and socioeconomic correlates within a single ecological framework.

## 2. Materials and Methods

### 2.1. Study Design and Setting

We conducted a cross-sectional ecological study of inpatient care self-sufficiency across the 11 SMAs of Aichi Prefecture, Japan, for FY2024 (1 April 2024 to 31 March 2025). Aichi is a centrally located industrial prefecture of approximately 7.5 million inhabitants (2020 Census) which includes Japan’s third-largest metropolitan area and surrounding rural districts, with a 135-fold within-prefecture gradient in population density and substantial heterogeneity in population aging (Supplementary Table S1). Aichi Prefecture was selected as the study site for three reasons. First, this internal heterogeneity reproduces within a single prefecture the urban–rural contrast that motivates regional inpatient planning nationally, while holding the prefectural policy environment constant. Second, an established prefecture-wide DPC data collection platform (Section 2.2) enabled hospitals in all 11 SMAs to be covered under a single research protocol, which is rarely feasible across multiple prefectures. Third, with 11 SMAs, Aichi contains more planning areas than the national average of approximately seven SMAs per prefecture, providing comparatively many ecological units within one prefecture. The analytic unit was the SMA-by-MDC-by-surgical-stratum cell (*n* = 11 × 18 × 2 = 396), and the SMA-level mean self-sufficiency rate across the 36 MDC × surgical-stratum cells (*n* = 11) was the unit of inference for the primary model. The study followed the small-area variation tradition pioneered by Wennberg and colleagues [14] and the methodological principles of ecological study design [29]. Andersen’s predisposing–enabling–need taxonomy [30,31], including its area-level applications [32], was used only as a descriptive heuristic for organizing the explanatory variables; area-level coefficients were not interpreted as estimates of individual-level Andersen parameters. The study followed the STROBE reporting guideline for observational studies [33].

### 2.2. Data Sources

**Figure 1:**
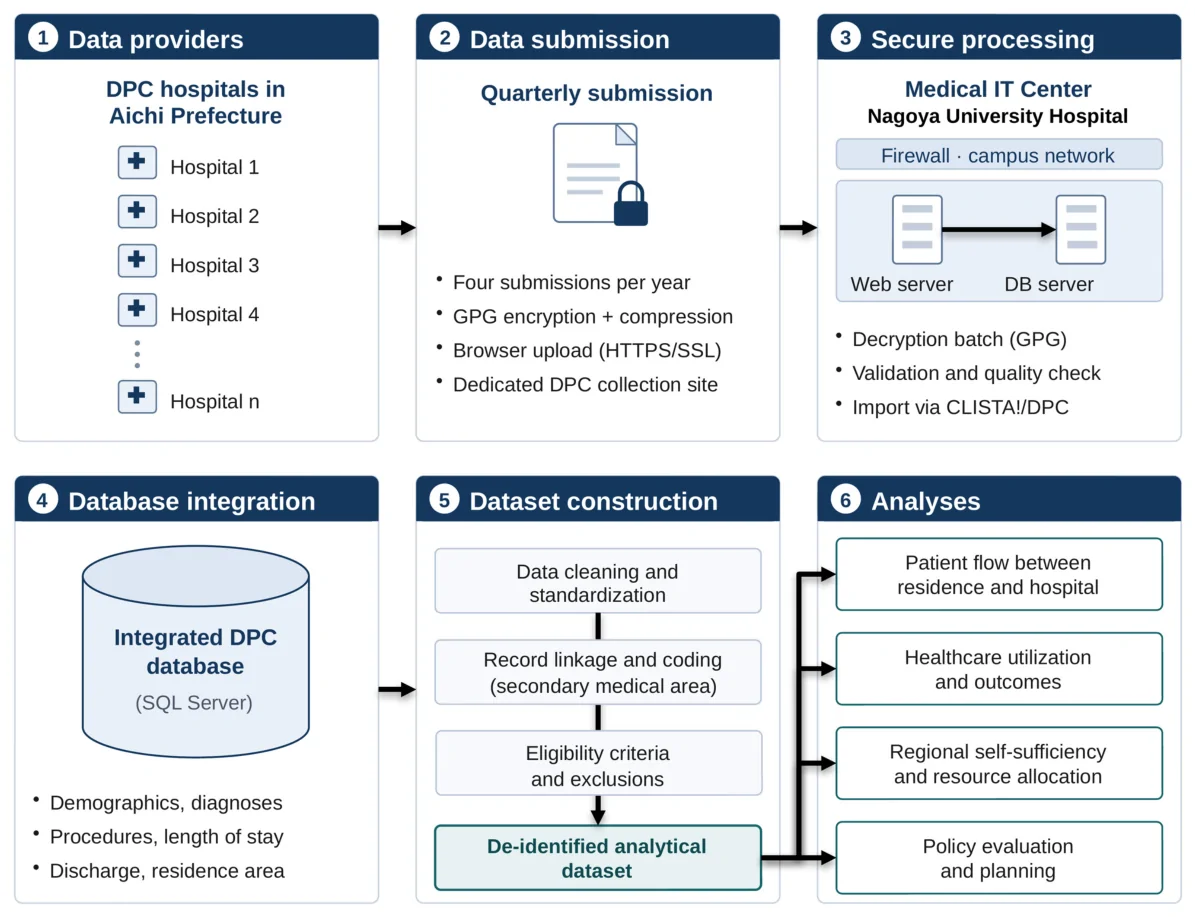
Overview of the DPC data collection platform and construction of the analytical dataset. Participating DPC hospitals in Aichi Prefecture submit encrypted DPC files quarterly through a dedicated collection site to the Medical IT Center, Nagoya University Hospital, where the files are decrypted, validated, and imported into an integrated DPC database on a secure server. Analytical datasets are then constructed through data cleaning, record linkage and coding to secondary medical areas, and application of eligibility criteria, yielding de-identified datasets for research; the present study corresponds to the analysis of regional care self-sufficiency. DB, database; DPC, Diagnosis Procedure Combination; GPG, GNU Privacy Guard; HTTPS/SSL, hypertext transfer protocol secure/secure sockets layer.

Inpatient utilization data were extracted from the DPC Form-1 discharge records of 203 of the 276 DPC-participating acute-care hospitals in Aichi Prefecture (73.6%) that provided data for research use under the present protocol during FY2024; the participating hospitals covered all 11 SMAs. The data collection and management workflow is summarized in Figure 1. Participating hospitals submitted encrypted DPC files quarterly through a dedicated collection site; the files were decrypted, validated, and imported into an integrated database on a secure server, from which the de-identified analytic dataset was constructed. The DPC Form-1 records each admission’s patient residence (at the SMA level), primary diagnosis, MDC (18 categories), and the presence of surgical procedures with K-code fees, permitting reconstruction of resident-by-admitting-SMA flow matrices for each MDC × surgical-stratum cell. The DPC dataset was assembled under the present research protocol and is not publicly distributed.

Public denominators for the explanatory variables were extracted from Japanese government statistics, with each indicator drawn from its appropriate source: (i) the proportion of the population aged ≥75 years and the household-composition variables were taken from the 2020 Population Census of Japan [34]; (ii) physician density was taken from the 2022 Survey of Physicians, Dentists and Pharmacists conducted by the Ministry of Health, Labour and Welfare (MHLW) [35]; (iii) hospital density, general-bed density, and psychiatric-bed density were taken from the FY2024 Survey of Medical Institutions and the Hospital Report (MHLW) [35]; (iv) cause-specific death counts (the numerators of the standardized mortality ratios, SMRs) were taken from the Vital Statistics in Japan (MHLW; computation procedure described in Section 2.4) [36]; (v) the areas (km^2^) used as the denominator of the supply-density indicators, together with the Rook-contiguity adjacency between SMAs, were derived from the Digital National Land Information of the Geospatial Information Authority of Japan [37]; and (vi) the welfare-benefit recipient rate and long-term-care certification rate were taken from 2024 Aichi prefectural administrative releases [38]. All explanatory variables were aggregated to the SMA level. Because Japanese government statistics differ in release cycles, each indicator was drawn from the most recent official release available at the time of analysis: the Population Census is quinquennial (2020 is the latest), the Survey of Physicians, Dentists and Pharmacists is biennial (2022 is the latest published), and the facility and bed statistics are compiled annually (FY2024, matching the outcome window). These explanatory variables describe structural characteristics of areas that change slowly over time, so we assume that the modest differences in reference years do not materially affect the cross-sectional area-level comparisons, although this assumption cannot be tested directly.

### 2.3. Outcome Variables

The primary outcome was the care self-sufficiency rate (%), defined for each SMA × MDC × surgical-stratum cell as the proportion of inpatients residing in the SMA who were admitted to hospitals within the same SMA, computed according to the following equation:

Self-sufficiency rate*_i_*_,*k*,*s*_ = (*N*_intra,*i*,*k*,*s*_/*N*_total,*i*,*k*,*s*_) × 100

where *N*_intra,*i*,*k*,*s*_ is the number of inpatients residing in SMA *i* who were admitted to hospitals within the same SMA *i* for MDC *k* and surgical stratum *s* (0 = non-surgical, 1 = surgical), and *N*_total,*i*,*k*,*s*_ is the total number of inpatients residing in SMA *i* for that cell, regardless of admitting SMA. Throughout, these flow indicators are computed within the participating DPC hospital network (203 of the 276 DPC acute-care hospitals; Section 2.2): admissions to non-participating hospitals or to hospitals outside the prefecture are not observed, so the self-sufficiency rate quantifies resident-to-provider flow within this network rather than complete residence-based inpatient utilization (see the coverage limitation in Section 4.8).

**Figure 2:**
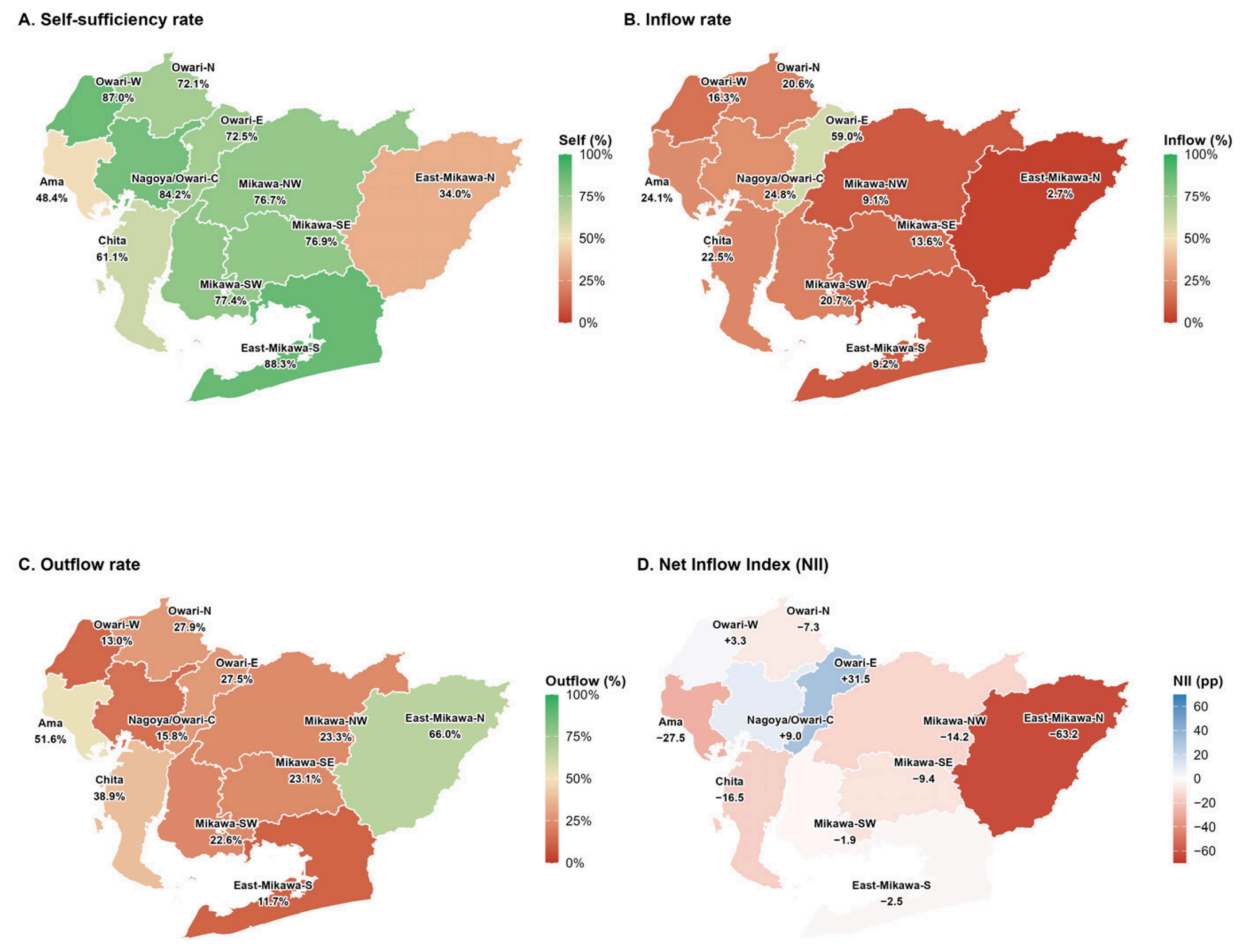
Inpatient care self-sufficiency and flow indicators. Geographic distribution of inpatient care self-sufficiency (**A**), inflow (**B**), outflow (**C**), and Net Inflow Index (**D**) across the 11 secondary medical areas of Aichi Prefecture, FY2024. Each panel shows the SMA-level mean across 18 Major Diagnostic Categories in the non-surgical stratum. MDC, Major Diagnostic Category; NII, Net Inflow Index; SMA, secondary medical area. SMA abbreviations: Nagoya/Owari-C, Nagoya/central Owari; Ama, Ama; Owari-E, eastern Owari; Owari-W, western Owari; Owari-N, northern Owari; Chita, Chita peninsula; Mikawa-NW, northwestern Mikawa; Mikawa-SW, southwestern Mikawa; Mikawa-SE, southeastern Mikawa; East-Mikawa-N, northern east Mikawa; East-Mikawa-S, southern east Mikawa.

**Figure 3:**
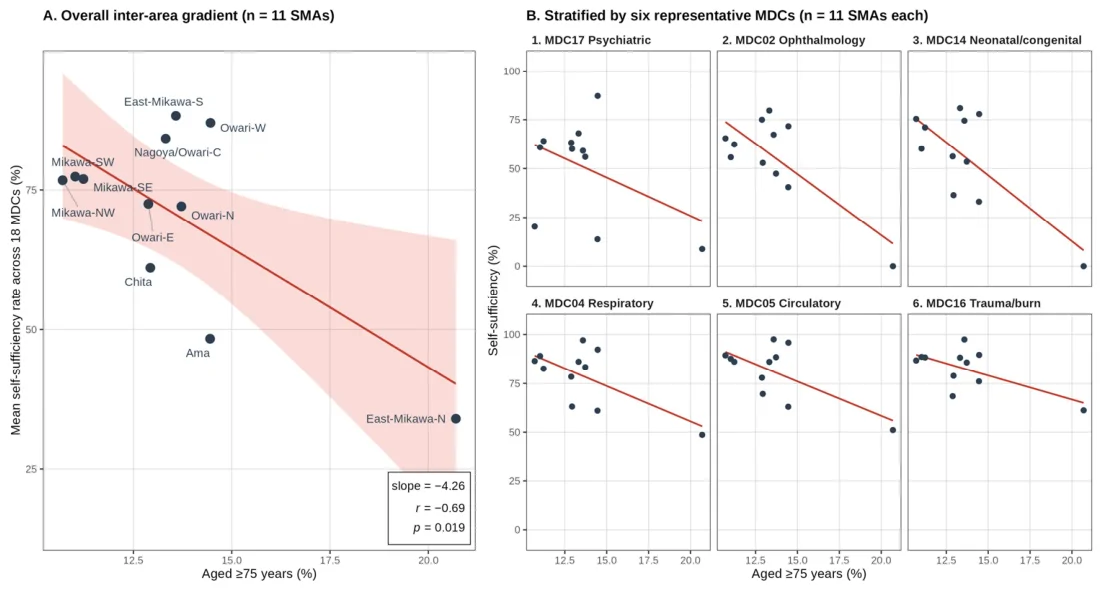
Population aging and inpatient care self-sufficiency. Inter-area gradient of inpatient care self-sufficiency by population aging across the 11 secondary medical areas of Aichi Prefecture. (**A**) Overall gradient (*n* = 11 SMAs). (**B**) Stratified by six representative Major Diagnostic Categories. The straight line indicates the least-squares regression; slope, Pearson correlation coefficient (r), and *p*-value are displayed in Panel A. The shaded area indicates the 95% confidence interval of the regression line. MDC, Major Diagnostic Category; SMA, secondary medical area.

Three additional flow indicators were computed descriptively for the SMA-level summary (Table 1 and Figure 2) but were not used for inferential modeling: the outflow rate (mathematical complement, outflow = 100 − self-sufficiency); the inflow rate (admissions in an SMA’s hospitals contributed by residents of other SMAs, divided by total admissions in that SMA); and the Net Inflow Index (NII; inflow rate minus outflow rate, in percentage points). For the inferential models (Table 2), the SMA-level mean self-sufficiency rate was computed across all 36 MDC × surgical-stratum cells; for the descriptive displays (Table 1, Figure 2, and Figure 3, Panel A), the 18 non-surgical cells per SMA were averaged. In summary, the study used four flow indicators: self-sufficiency (the primary outcome, with all observed admissions of residents of each SMA in the corresponding MDC × surgical stratum within the participating DPC hospital network as its denominator), outflow (its mathematical complement within the same observed network), inflow (with admissions to hospitals located in each SMA as its denominator), and the NII; their definitions, denominators, and roles are tabulated in Supplementary Table S2.

### 2.4. Explanatory Variables

Explanatory variables were organized into four blocks following Andersen’s descriptive taxonomy [30]. The demographic block included one variable: the proportion of the population aged ≥75 years. The supply block included four density variables (per km^2^): physician density, hospital density, general-bed density, and psychiatric-bed density. The need block, used as the pre-specified primary inferential set, included six cause-specific SMRs: cerebrovascular disease, ischemic heart disease, pneumonia, chronic obstructive pulmonary disease (COPD), all-cancer, and liver disease. An exploratory analysis (Table 3) extended this set to all 18 cause-specific SMRs available in the Vital Statistics in Japan. The socioeconomic block included five variables: the welfare-benefit recipient rate (per 1000 population), the proportion of elderly single-person households, the proportion of elderly couple-only households, the proportion of households with at least one elderly member, and the long-term-care certification rate. The definition, unit, reference year, and data source of every outcome and explanatory variable are summarized in Supplementary Table S2.

Cause-specific SMRs were computed by indirect standardization using the 2015 national age- and sex-specific cause-specific death rates as the reference, with observed deaths summed over the 5-year pool 2020–2024, as follows:

SMR_i,c_ = (Σ_t = 2020–2024_ *D*_obs,i,c,t_)/(Σ_t = 2020–2024_ Σ_a,sex_ *r*_c,a,sex,2015_ × *P*_i,a,sex,t_) × 100

where *D*_obs,i,c,t_ is the observed number of deaths in SMA *i* attributable to cause *c* in year *t*; *r*_c,a,sex,2015_ is the 2015 national reference death rate for cause *c*, age group *a*, and sex; and *P*_i,a,sex,t_ is the population in SMA *i* by age group *a* and sex in year *t*. An SMR of 100 indicates parity with the 2015 national reference; values above 100 indicate higher-than-expected mortality.

All explanatory variables were z-standardized across the 11 SMAs (mean 0, SD 1) before regression analysis to facilitate comparability of standardized coefficients (β) across variables on different scales.

### 2.5. Statistical Analysis

Because the number of SMAs in Aichi Prefecture is limited to 11, the primary inferential analysis was deliberately specified as a series of pre-specified single-predictor ecological regressions rather than a fully adjusted multi-predictor model, in order to avoid over-fitting and unstable estimates that would arise from simultaneously entering many predictors at this aggregation level. Effect-size consistency across model specifications, rather than fully adjusted multi-predictor estimation, was the pre-specified focus of inference. For each predictor, an ordinary least squares regression at the SMA level was fitted (Model A, *n* = 11), with the SMA-level mean self-sufficiency rate across the 36 cells as the dependent variable; the standardized regression coefficient (β), its 95% confidence interval, and the two-sided *p*-value were reported. Because the surgical and non-surgical values arise from the same SMA × MDC combinations and are therefore related rather than independent, the difference in cell-level self-sufficiency between the two strata was tested with a paired *t*-test across the 198 SMA × MDC pairs, with a Wilcoxon signed-rank test as a non-parametric sensitivity analysis.

Two pre-specified sensitivity analyses used the same single-predictor structure. First, a spatial-lag-X (SLX) specification (Model B, *n* = 11) added the row-standardized Rook-contiguity neighbor average of the focal predictor as an additional regressor, to assess the influence of spatial autocorrelation; Moran’s I (row-standardized Rook weights, permutation test) was computed on the Model A residuals. Second, a cell-level binomial logistic regression (Model C, *n* = 396) used a binary cell-level outcome (self-sufficiency above the median of the corresponding MDC × surgical stratum = 1) and the same predictor, adjusted for surgical stratum, with SMA cluster-robust standard errors computed via the sandwich estimator [39].

For the exploratory screening of 18 cause-specific SMRs (Table 3), separate mixed-effects linear regression models were fitted with crossed random intercepts for SMA and MDC, using the lme4 (version 2.0.6) and lmerTest (version 3.2.1) packages [40,41] with Satterthwaite-approximated degrees of freedom; 95% confidence intervals for these models were computed from t-quantiles based on the same Satterthwaite degrees of freedom. Because these cause-specific SMRs were expected to be correlated and the analysis was intended to describe disease-specific patterns rather than to define primary findings, nominal *p*-values were presented without formal multiplicity correction such as false-discovery-rate control [42]; the findings were interpreted as exploratory and hypothesis-generating, and primary inference for the cause-specific need block was restricted to the six pre-specified SMRs reported in Table 2. A null mixed-effects model (intercepts only) with crossed random intercepts for SMA and MDC was used to estimate variance components and the intra-class correlation coefficient at the SMA level (ICC_SMA_). A two-sided *p*-value less than 0.05 was defined as statistically significant for the pre-specified primary analyses. All statistical analyses were conducted using R, version 4.6.0 (R Foundation for Statistical Computing, Vienna, Austria).

### 2.6. Ethics

The study protocol was approved by the Clinical Research Review Committee of Nagoya University (Approval No. 2022-0203-4; 3 February 2022). The study analyzed aggregated, de-identified institutional records from the DPC Form-1 dataset; the requirement for individual informed consent was waived in accordance with the Japanese Ethical Guidelines for Medical and Health Research Involving Human Subjects.

## 3. Results

### 3.1. Variation in Inpatient Care Self-Sufficiency Across the 11 Secondary Medical Areas

Characteristics of the 11 SMAs are summarized in Supplementary Table S1. Table 1 shows the care self-sufficiency rates by MDC across the 11 SMAs. Across the 18 non-surgical MDC cells per SMA (*n* = 198), the mean self-sufficiency rate varied widely between SMAs, ranging from 34.0% in East-Mikawa-N (the most rural SMA; area 1159 km^2^; 20.7% aged ≥75 years) to 88.3% in East-Mikawa-S. At the cell level (*n* = 396), self-sufficiency was 8.5 percentage points lower for surgical than for non-surgical cells (paired *t*-test across the 198 SMA × MDC pairs: 95% CI of the difference 5.9 to 11.1 percentage points, *p* < 0.001; Wilcoxon signed-rank test, *p* < 0.001). Net outflow patterns dominated specialty-concentrated and life-stage MDCs (NII < −10 percentage points for MDC02 Ophthalmology, MDC08 Skin, MDC09 Breast, MDC12 Female reproductive, MDC13 Blood/immune, MDC14 Neonatal/congenital, and MDC17 Psychiatric), whereas chronic high-prevalence MDCs (MDC04 Respiratory, MDC05 Circulatory, MDC06 Digestive) showed mean self-sufficiency exceeding 75% with NII between −4 and −7 percentage points (Table 1).

### 3.2. Geographic Distribution of Self-Sufficiency, Inflow, Outflow, and Net Inflow Index

Figure 2 shows the SMA-level mean values of the four flow indicators across the 11 SMAs. The self-sufficiency map (Figure 2, Panel A) showed substantial geographic variation, with values ranging from 34.0% in the rural eastern mountain SMA (East-Mikawa-N) to 88.3% in East-Mikawa-S, and high values also in the densely populated central plain (Owari-W 87.0%, Nagoya/Owari-C 84.2%). The outflow map (Panel C) was the exact complement of the self-sufficiency map (outflow = 100 − self-sufficiency by construction), whereas the inflow rate (Panel B) uses the patients treated within each SMA as its denominator and therefore carries separate information. The Net Inflow Index (Panel D) further accentuated the contrast: East-Mikawa-N had the most negative NII (−63.2 percentage points), indicating substantial net outflow, whereas Nagoya/Owari-C (+9.0) and Owari-E (+31.5) had the most positive NII values, consistent with their tertiary-referral roles for surrounding areas.

### 3.3. Inter-Area Gradient by Population Aging

Figure 3 shows the inter-area gradient of mean self-sufficiency against the proportion of the population aged ≥75 years. The overall regression (Panel A, *n* = 11 SMAs) yielded a slope of −4.26 percentage points per percentage-point increase in the share of those aged ≥75 years (Pearson r = −0.69, *p* = 0.019), indicating a negative ecological association at the SMA level. In the descriptive stratification by six representative MDCs (Panel B), the negative slopes appeared steeper for specialty-concentrated MDCs (MDC02 Ophthalmology and MDC14 Neonatal/congenital) and shallower for chronic-care MDCs (MDC04 Respiratory and MDC05 Circulatory); no formal between-MDC comparison of slopes was performed.

### 3.4. Pre-Specified Single-Predictor Ecological Associations

Table 2 shows the results of the pre-specified single-predictor ecological associations (Model A, region-level OLS, *n* = 11; each predictor entered separately to avoid over-fitting at this aggregation level). A one-standard-deviation increase in the SMA share of residents aged ≥75 years was associated with a 12.80 percentage-point lower self-sufficiency (β = −12.80; 95% CI −22.84 to −2.75; *p* = 0.018). Among the six pre-specified cause-specific SMRs in the need block (the pre-specified primary set), cerebrovascular disease SMR was the strongest negative correlate (β = −12.72; 95% CI −22.82 to −2.62; *p* = 0.019); the other five SMRs (ischemic heart disease, pneumonia, COPD, all-cancer, liver disease) did not reach significance in Model A. Within the socioeconomic block, the proportion of households with at least one elderly member was the most negative single-predictor correlate (β = −13.53; 95% CI −23.02 to −4.05; *p* = 0.010). Supply-density variables in the supply block showed consistently positive but non-significant point estimates in Model A (β +7.93 to +8.47; *p* = 0.156–0.188), as expected given the limited statistical power at *n* = 11.

The two pre-specified sensitivity analyses showed similar directions and magnitudes for the key single-predictor associations. The spatial-lag-X model (Model B, *n* = 11) yielded coefficients of similar sign and magnitude (aged ≥75 years β = −11.14, *p* = 0.027; cerebrovascular SMR β = −13.18, *p* = 0.012; ≥1 elderly household β = −12.75, *p* = 0.008), with Moran’s I on the Model A residuals (I = −0.25, *p* = 0.48) indicating no significant residual spatial autocorrelation. The cell-level binomial logistic regression with SMA cluster-robust standard errors (Model C, *n* = 396) showed that three of the four supply-density variables (physician, general-bed, and psychiatric-bed densities) were associated with above-median self-sufficiency (odds ratios 2.51–3.16; all *p* < 0.05), whereas hospital density had a positive but non-significant estimate (OR 3.71; *p* = 0.080). Liver-disease SMR was the strongest positive predictor in Model C (OR 3.72; 95% CI 1.54–9.02; *p* = 0.004), and the welfare-benefit recipient rate also reached cluster-robust significance (OR 2.63; 95% CI 1.16–5.94; *p* = 0.021).

### 3.5. Exploratory Screening of 18 Cause-Specific SMRs

Table 3 shows the results of the exploratory screening of all 18 cause-specific SMRs (cell-level n = 396 with crossed random intercepts for SMA and MDC). The screening suggested a disease-specific pattern, with the strongest nominal negative associations observed for cerebrovascular disease SMR (β = −12.14; 95% CI −21.79 to −2.50; *p* = 0.019) and cerebral infarction SMR (β = −12.61; 95% CI −21.91 to −3.31; *p* = 0.013), and positive point estimates for several chronic-care-related mortality indicators. Stomach cancer SMR (β = +10.26, *p* = 0.060) and COPD SMR (β = −9.26, *p* = 0.097) reached only marginal significance. In terms of the direction of point estimates only, negative values were observed for several time-sensitive acute conditions (cerebrovascular disease, cerebral infarction, COPD, acute myocardial infarction) and positive values for several chronic-care-related burdens (liver disease, stomach cancer, pneumonia, senility); this grouping is interpretive and was not formally tested. As pre-specified, no formal multiplicity correction was applied to these nominal *p*-values, and the screening findings are interpreted as exploratory and hypothesis-generating; primary inference is restricted to the six pre-specified SMRs in Table 2. The variance components from a null mixed-effects model yielded σ^2^(SMA) = 335.8 (54%), σ^2^(MDC) = 87.3 (14%), and σ^2^(residual) = 197.0 (32%), with ICC_SMA_ = 0.54, indicating that the SMA level accounts for substantial unexplained between-area variation.

## 4. Discussion

### 4.1. Principal Findings

This study described inter-area variation in inpatient care self-sufficiency across the 11 SMAs of Aichi Prefecture, Japan, for FY2024, and examined its area-level correlates. Four findings emerged. First, self-sufficiency varied widely across planning areas (34.0–88.3%), with the SMA level accounting for 54% of cell-level variance. Second, population aging and the proportion of households containing at least one elderly member were the strongest negative correlates. Third, supply density showed consistently positive associations across model specifications. Fourth, an exploratory 18-cause screening suggested an acute–chronic contrast in disease-specific care concentration. To our knowledge, this is the first study to quantify disease-stratified inpatient care self-sufficiency across all SMAs of a Japanese prefecture and to examine its demographic, supply, need, and socioeconomic correlates within a single ecological framework. Because individual-level case-mix data were not available, none of these associations is adjusted for patient-level severity or comorbidity; this constraint applies to all results and is discussed in Section 4.7 and Section 4.8.

### 4.2. Population Aging, Household Composition, and Self-Sufficiency

A possible reason for the negative association between the proportion of the population aged ≥75 years and care self-sufficiency (β = −12.80; 95% CI −22.84 to −2.75) is that older populations tend to reside in lower-density rural SMAs where local hospital capacity is limited and patients with specialty needs are commonly referred to higher-volume urban centers, consistent with prior Japanese work showing that patients in less-resourced areas travel to better-equipped metropolitan hospitals and that aging areas face sparser specialty resources [20,21] and with broader Japanese evidence linking area-level socioeconomic position to mortality and healthcare access [27,28]. A novel finding was that the proportion of households containing at least one elderly member was the strongest negative single-predictor correlate (β = −13.53; 95% CI −23.02 to −4.05), even when entered separately from the share of those aged ≥75 years and the cause-specific SMRs. The contrasting odds for elderly single-person households (Model C OR 1.73; 95% CI 0.91–3.28) and elderly couple-only households (OR 0.57; 95% CI 0.28–1.16), although neither interval excluded unity, raise the possibility that living arrangement, rather than population age alone, is related to how local inpatient care is used; the present ecological data do not establish how living arrangements influence hospital choice, and these household-type differences warrant individual-level investigation. These results are consistent with prior Japanese work on household context and healthcare utilization among older adults [43,44], and suggest that aggregate aging measures alone may miss important heterogeneity.

### 4.3. Disease-Specific Patterns of Care Concentration

The exploratory 18-cause screening suggested a contrast in the direction of coefficients: time-sensitive acute conditions (cerebrovascular disease, cerebral infarction, COPD, acute myocardial infarction) consistently showed negative point estimates, whereas chronic-care-amenable burdens (liver disease, stomach cancer, pneumonia, senility) showed positive ones. The strongest nominal negative associations, observed for cerebrovascular disease SMR (β = −12.14, *p* = 0.019) and cerebral infarction SMR (β = −12.61, *p* = 0.013), are clinically plausible given the time-sensitive and regionally concentrated nature of acute stroke care: SMAs with a higher cerebrovascular mortality burden may include populations whose stroke events disproportionately bypass local hospitals in favor of higher-volume comprehensive stroke centers, particularly where endovascular or neurosurgical capacity is concentrated, and nationwide Japanese evidence shows that greater comprehensive stroke-center capabilities are associated with lower in-hospital stroke mortality [45]. Conversely, the strongest positive cause-specific association was for liver-disease SMR in the cell-level model (Model C OR 3.72; 95% CI 1.54–9.02), which is plausible if liver-related care, such as longitudinal management of chronic hepatitis, cirrhosis, and hepatocellular carcinoma, is locally retained even in SMAs with elevated liver-disease mortality. Because the screening was exploratory and reported without formal multiplicity correction, this apparent acute–chronic contrast should be regarded as a hypothesis-generating signal to be tested with greater statistical power (for example, multi-prefecture pooling); consistent with this, the magnitude of small-area variation is known to differ systematically across conditions and procedures [16], and geographic mortality patterns in Japan likewise differ by cause [26].

### 4.4. Healthcare Supply Density

The four supply-density variables showed positive associations with care self-sufficiency in all model specifications, although they did not reach nominal significance in the primary Model A (*n* = 11; β +7.93 to +8.47; *p* = 0.156–0.188). In the cell-level Model C with cluster-robust standard errors, three of the four supply variables (physician, general-bed, and psychiatric-bed densities) were associated with above-median self-sufficiency at *p* < 0.05 (odds ratios 2.51–3.16). Although higher supply density plausibly supports higher self-sufficiency, each supply variable was entered separately, and the non-significant Model A estimates most likely reflect the limited precision available at *n* = 11; the consistency in sign and magnitude across Models A, B, and C nevertheless supports the supply–outcome association.

### 4.5. Comparison with Other Health Systems and International Applicability

The present patterns are consistent with small-area variation research in other health systems. The original observation that utilization varies across small areas beyond what population need explains was made in US hospital service areas [14,15] and has since been confirmed across OECD countries [16]. In South Korea, spatial accessibility to healthcare services is markedly lower in rural, mountainous, and coastal areas, and disadvantaged groups experience compounded barriers [46]; in Japan, sub-prefectural analyses have shown that the determinants of healthcare expenditure differ even between adjacent central cities and suburbs [23], and nationwide analyses have documented patient flow from less-resourced to metropolitan areas [20]. The present study adds a disease-stratified flow dimension to this literature: because the SMA × MDC × surgical-stratum framework relies only on discharge records with residence coding, case-mix classification, and standard government statistics, it may also be applicable to other health systems that plan hospital capacity through sub-national regions and maintain comparable administrative data, and it gives planners condition-specific information that aggregate flow statistics cannot provide. Comparable sub-national planning units exist in many health systems, including hospital service areas and referral regions in the United States, integrated care system footprints in England, and regional health service areas in the Republic of Korea. In any of these settings, disease-stratified self-sufficiency can be computed from routine discharge abstracts that record patient residence and case-mix, allowing planners outside Japan to identify which service lines are regionally concentrated and which remain locally complete, and allowing researchers to test whether the aging and household-composition associations observed here generalize across systems.

### 4.6. Implications for Policy, Practice, and Research

The findings also have implications for Japanese health policy. FY2024, the outcome window of this study, is the first year of the Eighth Medical Care Plan (2024–2029), under which prefectures review SMA configurations and bed-function allocation [11], and restructuring of the delivery system toward 2040 is under national discussion [47]. Three descriptive implications follow from our results. First, for time-sensitive, regionally concentrated conditions such as acute stroke, uniform within-SMA completion targets may be less informative than indicators of inter-SMA referral networks, given that net outflow was concentrated in specialty and life-stage MDCs. Second, chronic high-prevalence MDCs (respiratory, circulatory, digestive) maintained mean self-sufficiency above 75% even in aging SMAs, suggesting that local completion capacity for these conditions should be monitored as populations age. Third, household composition, not only aggregate aging, covaried with self-sufficiency, suggesting that demand projections and bed-function reallocation could incorporate living-arrangement indicators alongside age structure. These implications are descriptive: they identify where planning attention may be warranted, not which reallocation is optimal. Beyond Japan, two further uses follow. For practice, health authorities that plan hospital capacity through sub-national regions can monitor disease-specific self-sufficiency as a routine indicator, so that decisions to concentrate services regionally are weighed against the travel burden they impose on aging populations. For research, the framework offers a replicable outcome definition for cross-system comparisons of care regionalization, and multi-region or individual-level extensions could test the hypothesis-generating disease-specific patterns reported here.

### 4.7. Causal Interpretation of Ecological Findings

The associations reported here should not be interpreted causally, for three reasons that are inherent to the design. First, all variables are area-level aggregates, so the associations describe SMAs, not individuals; inferring individual-level relationships from area-level coefficients risks the ecological fallacy, and the direction and magnitude of individual-level associations may differ from those observed here [29]. Second, the design is cross-sectional: the outcome and the explanatory variables refer to overlapping periods, so temporal precedence cannot be established, and reverse pathways (for example, established patient-flow patterns shaping local supply) cannot be excluded. Third, unmeasured area-level factors, including patient case-mix, referral customs, and historical hospital locations, may confound any of the observed associations. Accordingly, the estimates in Table 2 and Table 3 are correlational descriptions intended to inform planning attention and hypothesis generation, not estimates of intervention effects; causal evaluation would require individual-level linkage and designs with temporal separation between need measures and utilization outcomes.

### 4.8. Strengths and Limitations

A strength of this study is that institutional DPC inpatient records from 203 acute-care hospitals covering all 11 SMAs of Aichi Prefecture were combined with the Vital Statistics in Japan, the 2020 Population Census, the Survey of Physicians, Dentists and Pharmacists, and the Survey of Medical Institutions and the Hospital Report. The results therefore describe inpatient care self-sufficiency at the SMA level, the administrative unit at which Japanese inpatient-care planning is implemented, rather than relying on prefecture-level aggregates, which generally cannot capture within-prefecture heterogeneity. Although prior Japanese ecological work has separately described regional disparities in healthcare supply and in cause-specific mortality [21,22,27], this study integrated disease-stratified inpatient utilization with cause-specific SMRs and household-composition socioeconomic variables in a single 396-cell analytic dataset, enabling joint description across four domains (demographic, supply, need, and socioeconomic) that has rarely been attempted within a single Japanese prefecture. By using municipal-level Vital Statistics aggregated to the SMA level, the analysis also revealed disease-specific geographical patterns (e.g., the acute-stroke versus chronic-care contrast) that prefecture-level analyses would necessarily obscure.

This research has some limitations. First, as elaborated in Section 4.7, this is an ecological study, and the possibility of ecological fallacy cannot be excluded [29]; inferences refer to area-level patterns and cannot be extended to individual-level mechanisms. Second, the cross-sectional design precludes causal inference, and the FY2024 outcome window partially overlaps with the 2020–2024 SMR pool used for the need block. Because the SMRs are 5-year pooled, age-standardized measures of structural mortality patterns rather than annual outcomes, this partial overlap is unlikely to induce mechanistic circularity, but a strictly pre-temporal need measure could not be evaluated in the present dataset. A pre-period need measure, for example SMRs pooled over 2015–2019, would require an additional extraction from the Vital Statistics and is a priority for future work. Third, the analytic sample is restricted to one prefecture (Aichi) with *n* = 11 SMAs, and the deliberate single-predictor specification of the primary analysis, chosen to avoid over-fitting at this aggregation level, precludes fully adjusted multivariable estimation; effect-size consistency across three model specifications partially mitigates but does not eliminate this limitation. Fourth, individual-level patient case-mix data (admission severity, comorbidity, primary insurance type) were not available in the DPC Form-1 aggregate dataset; differences in case-mix between SMAs may confound the supply–outcome association. Fifth, educational attainment was not included as a socioeconomic variable, as municipal-level shares were not consistently available. Sixth, the 18-cause SMR screening is reported with nominal *p*-values without formal multiplicity correction and is interpreted as exploratory. Seventh, because the 2020–2024 SMR pool spans the COVID-19 pandemic, pandemic-related changes in mortality patterns and cause-of-death certification may have affected the cause-specific SMRs. Potential pathways include time-varying excess mortality, changes in certification and coding (particularly when COVID-19 occurred with pneumonia), and indirect effects of altered care-seeking and healthcare delivery on non-COVID causes. Pooling five years may attenuate isolated annual fluctuations but cannot eliminate a geographically differential multi-year pandemic effect. COVID-19 itself was not included among the 18 analyzed causes; therefore, if the magnitude or timing of pandemic effects differed across SMAs, cause-specific SMRs could have been differentially affected. Associations involving pandemic-sensitive causes, particularly pneumonia and senility, should therefore be interpreted with caution. Eighth, the DPC dataset comprises records from the 203 hospitals (of 276 DPC acute-care hospitals in Aichi Prefecture; 73.6%) that agreed to provide data; admissions to the 73 non-participating hospitals, and to hospitals outside the prefecture, are not captured. The self-sufficiency metric therefore reflects resident-to-provider flow observed within the participating Aichi DPC hospital network rather than residence-based inpatient utilization in absolute terms, and outflow may be undercaptured both where non-participating hospitals are located and in SMAs along the prefectural border (notably East-Mikawa-N, East-Mikawa-S, Mikawa-NW, and parts of Owari-W). Because hospital participation was not random with respect to geography, the magnitude and direction of this coverage-related bias cannot be determined from the present data. Further research using individual-level data (in particular, linkage of DPC discharge records with patient-level case-mix information) and extension to multi-prefecture pooling would clarify whether the associations observed at the SMA level persist after individual-level adjustment, enhance generalizability, and permit fully adjusted multi-predictor estimation; cause-specific analyses of the cerebrovascular findings, ideally with stroke-registry data, would further illuminate the acute-stroke-care concentration pattern observed here.

## 5. Conclusions

This ecological study described substantial inter-area variation in inpatient care self-sufficiency across the 11 secondary medical areas of Aichi Prefecture and characterized its area-level correlates using DPC discharge records linked to Japanese government statistics. Population aging, household composition, supply density, and cause-specific mortality patterns were each associated with care self-sufficiency at the SMA level, and an exploratory acute–chronic contrast in disease-specific care concentration was suggested. These findings suggest that small-area inpatient planning could distinguish conditions suited to regional concentration from those requiring local completion, and consider household structure alongside aggregate aging indicators. Confirmation with individual-level data and extension to multiple prefectures are needed before these ecological correlates can inform healthcare resource allocation policies.

## Figures and Tables

**Table 1:** Inpatient care self-sufficiency and flow indicators by Major Diagnostic Category.

MDC	Diagnostic Category	Self-Sufficiency, Mean (SD), Range	Outflow, Mean (SD), Range	Inflow, Mean (SD), Range	NII, Mean
**G1: Chronic, high-prevalence**					
MDC04	Respiratory	78.9 (15.0), 48.6–97.1	21.1 (15.0), 2.9–51.4	15.7 (13.3), 5.3–52.3	−5.4
MDC05	Circulatory	81.2 (14.4), 51.1–97.5	18.8 (14.4), 2.5–48.9	15.2 (13.4), 4.1–52.5	−3.6
MDC06	Digestive	75.7 (13.9), 48.9–92.4	24.3 (13.9), 7.6–51.1	17.6 (12.9), 4.1–50.6	−6.7
MDC10	Endocrine/metabolic	78.7 (12.3), 52.9–94.6	21.3 (12.3), 5.4–47.1	16.2 (13.6), 3.5–53.9	−5.1
MDC11	Renal/urinary	78.3 (13.1), 52.8–95.9	21.7 (13.1), 4.1–47.2	17.4 (13.1), 7.3–51.9	−4.3
**G2: Acute, time-critical**					
MDC01	Nervous system	73.2 (18.6), 25.6–92.5	26.8 (18.6), 7.5–74.4	19.5 (15.1), 1.9–60.2	−7.3
MDC03	ENT	73.6 (13.7), 45.8–91.2	26.4 (13.7), 8.8–54.2	17.8 (15.5), 5.2–57.2	−8.6
MDC16	Trauma/burn	82.7 (10.6), 61.2–97.5	17.3 (10.6), 2.5–38.8	16.2 (9.4), 5.6–40.4	−1.1
**G3: Specialty-concentrated**					
MDC02	Ophthalmology	56.2 (22.1), 0.0–79.7	43.8 (22.1), 20.3–100.0	22.3 (22.0), 0.0–69.2	−21.5
MDC07	Musculoskeletal	66.4 (16.0), 36.2–85.6	33.6 (16.0), 14.4–63.7	25.1 (18.2), 0.0–64.3	−8.4
MDC08	Skin	68.7 (20.4), 24.4–93.2	31.3 (20.4), 6.8–75.6	18.9 (17.2), 4.5–60.7	−12.3
MDC09	Breast	71.9 (23.6), 12.7–92.7	28.1 (23.6), 7.3–87.3	16.2 (17.6), 0.0–55.1	−11.8
MDC13	Blood/immune	66.1 (22.9), 21.2–90.6	33.9 (22.9), 9.4–78.8	21.7 (18.0), 0.0–63.4	−12.2
**G4: Life-stage**					
MDC12	Female reproductive	65.0 (27.4), 0.0–88.4	35.0 (27.4), 11.6–100.0	23.3 (17.6), 0.0–67.3	−11.7
MDC14	Neonatal/congenital	56.3 (24.7), 0.0–81.0	43.7 (24.7), 19.0–100.0	31.8 (21.3), 0.0–72.6	−12.0
MDC15	Pediatric	69.5 (28.1), 0.0–92.3	30.5 (28.1), 7.7–100.0	21.7 (17.1), 0.0–54.4	−8.8
**G5: Behavioral/social**					
MDC17	Psychiatric	51.0 (25.1), 8.8–87.3	49.0 (25.1), 12.7–91.2	32.0 (20.5), 0.0–77.7	−17.0
MDC18	Others	80.5 (11.7), 60.4–93.6	19.5 (11.7), 6.4–39.6	15.8 (14.8), 5.6–57.8	−3.7

Values are mean (SD) and range across the 11 secondary medical areas in the non-surgical stratum (*n* = 198 cells), expressed in %. The Net Inflow Index (NII) is expressed in percentage points (inflow − outflow). The outflow rate is the mathematical complement of self-sufficiency (outflow = 100 − self). A priori MDC groups: G1, chronic high-prevalence; G2, acute time-critical; G3, specialty-concentrated; G4, life-stage; G5, behavioral/social. MDC, Major Diagnostic Category; SD, standard deviation.

**Table 2:** Pre-specified single-predictor ecological associations between care self-sufficiency and demographic, supply, need, and socioeconomic predictors.

Standardized Predictor (z-Score)	Model A: Region OLS (*n* = 11) ᵃ β (95% CI)	*p*	Model B: SLX Spatial (*n* = 11) β (95% CI)	*p*	Model C: Logistic (*n* = 396) ᵇ OR (95% CI)	*p*
**Demographic (predisposing)**						
Aged ≥75 years (%)	−12.80 (−22.84, −2.75) *	0.018	−11.14 (−20.65, −1.62) *	0.027	0.59 (0.29, 1.21)	0.149
**Supply (enabling)**						
Physician density, per km^2^	+7.93 (−4.66, +20.51)	0.188	+8.55 (−4.76, +21.87)	0.177	2.53 (1.13, 5.71) *	0.025
Hospital density, per km^2^	+8.22 (−4.26, +20.69)	0.171	+8.60 (−4.48, +21.69)	0.168	3.71 (0.85, 16.18) ^†^	0.080
General-bed density, per km^2^	+8.46 (−3.93, +20.84)	0.157	+9.06 (−3.95, +22.07)	0.147	3.16 (1.08, 9.22) *	0.036
Psychiatric-bed density, per km^2^	+8.47 (−3.91, +20.85)	0.156	+10.15 (−2.95, +23.25)	0.112	2.51 (1.19, 5.32) *	0.016
**Need (standardized mortality ratios)**						
Cerebrovascular disease SMR	−12.72 (−22.82, −2.62) *	0.019	−13.18 (−22.52, −3.83) *	0.012	0.42 (0.18, 1.00) ^†^	0.051
Ischemic heart disease SMR	−0.67 (−14.59, +13.25)	0.916	−0.48 (−15.27, +14.32)	0.943	0.92 (0.44, 1.92)	0.820
Pneumonia SMR	+7.22 (−5.60, +20.04)	0.235	+6.67 (−7.51, +20.85)	0.309	1.86 (0.77, 4.51)	0.171
COPD SMR	−9.70 (−21.55, +2.16) ^†^	0.097	−9.12 (−22.37, +4.13)	0.151	0.87 (0.38, 2.00)	0.736
All-cancer SMR	+4.40 (−9.13, +17.93)	0.481	+7.04 (−9.03, +23.11)	0.342	1.40 (0.46, 4.25)	0.551
Liver disease SMR	+9.15 (−2.96, +21.25)	0.122	+6.34 (−8.70, +21.37)	0.359	3.72 (1.54, 9.02) **	0.004
**Socioeconomic status**						
Welfare benefit recipient rate, per 1000 population	+6.50 (−6.54, +19.54)	0.289	+6.66 (−7.03, +20.34)	0.294	2.63 (1.16, 5.94) *	0.021
Elderly single-person household rate, %	+4.99 (−8.42, +18.41)	0.421	+5.53 (−9.19, +20.25)	0.411	1.73 (0.91, 3.28) ^†^	0.092
Elderly couple-only household rate, %	−1.18 (−15.08, +12.73)	0.853	−2.42 (−15.90, +11.06)	0.690	0.57 (0.28, 1.16)	0.121
Households with ≥1 elderly member, %	−13.53 (−23.02, −4.05) *	0.010	−12.75 (−21.11, −4.39) **	0.008	0.49 (0.19, 1.25)	0.136
Long-term care certification rate, %	−4.82 (−18.27, +8.63)	0.438	−4.52 (−19.07, +10.03)	0.494	1.01 (0.34, 2.99)	0.990

All predictors were z-standardized before model fitting. β = percentage-point change in self-sufficiency per 1 SD increase in the predictor (Models A, B); OR = odds ratio per 1 SD (Model C). ᵃ Model A: ordinary least squares at the SMA level (primary model). ᵇ Model C: cell-level binomial logistic regression (outcome = self-sufficiency above the median of the corresponding MDC × surgical stratum) with cluster-robust standard errors (SMA cluster), adjusted for surgical stratum. Each predictor was entered separately. ^†^ *p* < 0.10; * *p* < 0.05; ** *p* < 0.01.

**Table 3:** Mixed-effects regression of care self-sufficiency on 18 cause-specific standardized mortality ratios (exploratory screening).

Cause-of-Death SMR (z-Score)	β (per 1 SD)	95% CI	*p*
All-cause SMR	−4.74	(−17.55, +8.07)	0.424
All-cancer SMR	+4.20	(−8.72, +17.11)	0.481
Stomach cancer SMR	+10.26 ^†^	(−0.55, +21.08)	0.060
Colorectal cancer SMR	−4.97	(−17.73, +7.79)	0.401
Liver/bile-duct cancer SMR	−1.11	(−14.38, +12.17)	0.855
Lung cancer SMR	+4.95	(−7.81, +17.72)	0.403
Heart disease SMR	−4.84	(−17.63, +7.95)	0.414
Acute MI SMR	−6.08	(−18.56, +6.41)	0.299
Ischemic heart disease SMR	−0.64	(−13.93, +12.65)	0.916
Cerebrovascular SMR	−12.14 *	(−21.79, −2.50)	0.019
Intracerebral hemorrhage SMR	−8.78	(−20.31, +2.76)	0.119
Cerebral infarction SMR	−12.61 *	(−21.91, −3.31)	0.013
Pneumonia SMR	+6.89	(−5.35, +19.13)	0.235
COPD SMR	−9.26 ^†^	(−20.58, +2.06)	0.097
Liver disease SMR	+8.73	(−2.82, +20.29)	0.122
Renal failure SMR	+4.70	(−8.12, +17.52)	0.428
Senility SMR	+5.43	(−7.22, +18.08)	0.357
Suicide SMR	+4.69	(−8.13, +17.51)	0.429

Mixed-effects linear regression with crossed random intercepts for SMA and MDC (*n* = 396 cells across surgical and non-surgical strata). β = percentage-point change in self-sufficiency per 1 SD increase in the standardized cause-specific SMR. *p* values and 95% CIs are based on Satterthwaite degrees of freedom. SMR, standardized mortality ratio; CI, confidence interval; MDC, Major Diagnostic Category; SMA, secondary medical area. ^†^ *p* < 0.10; * *p* < 0.05.

## Data Availability

The publicly available data sources used in this study (the 2020 Population Census, the Vital Statistics in Japan, the Survey of Physicians, Dentists and Pharmacists, the Survey of Medical Institutions and the Hospital Report, the Digital National Land Information, and the 2024 Aichi prefectural administrative releases) are listed in the References. The DPC Form-1 institutional records used to construct the analytic dataset are not publicly available; they are restricted under institutional data-sharing agreements with the 203 participating acute-care hospitals and the ethics approval from Nagoya University. Data are available from the corresponding author on reasonable request and with permission of the data-providing institutions.

## Supplementary Materials

The following supporting information can be downloaded at: https://www.mdpi.com/article/10.3390/healthcare14193322/s1, Table S1: Characteristics of the 11 secondary medical areas in Aichi Prefecture; Table S2: Definitions, units, reference years, and data sources of all outcome and explanatory variables.

## References

[1] United Nations, Department of Economic and Social Affairs, Population Division (2024). World Population Prospects 2024: Summary of Results.

[2] United Nations (2020). UN Decade of Healthy Ageing: Plan of Action 2021–2030.

[3] Beard J.R., Officer A., de Carvalho I.A., Sadana R., Pot A.M., Michel J.-P., Lloyd-Sherlock P., Epping-Jordan J.E., Peeters G.M.E.E., Mahanani W.R. (2016). The World report on ageing and health: A policy framework for healthy ageing. Lancet.

[4] Rudnicka E., Napierała P., Podfigurna A., Męczekalski B., Smolarczyk R., Grymowicz M. (2020). The World Health Organization (WHO) approach to healthy ageing. Maturitas.

[5] Organisation for Economic Co-operation and Development (2023). Health at a Glance 2023: OECD Indicators.

[6] Skou S.T., Mair F.S., Fortin M., Guthrie B., Nunes B.P., Miranda J.J., Boyd C.M., Pati S., Mtenga S., Smith S.M. (2022). Multimorbidity. Nat. Rev. Dis. Primers.

[7] World Health Organization (2015). World Report on Ageing and Health.

[8] Ikegami N., Yoo B.-K., Hashimoto H., Matsumoto M., Ogata H., Babazono A., Watanabe R., Shibuya K., Yang B.-M., Reich M.R. (2011). Japanese universal health coverage: Evolution, achievements, and challenges. Lancet.

[9] Sakamoto H., Rahman M.M., Nomura S., Okamoto E., Koike S., Yasunaga H., Kawakami N., Hashimoto H., Kondo N., Abe S.K. (2018). Japan Health System Review (Health Systems in Transition, Vol. 8, No. 1).

[10] Hashimoto H., Ikegami N., Shibuya K., Izumida N., Noguchi H., Yasunaga H., Miyata H., Acuin J.M., Reich M.R. (2011). Cost containment and quality of care in Japan: Is there a trade-off?. Lancet.

[11] Ministry of Health, Labour and Welfare (Japan) (2024). Outline of the Medical Care Planning System (Iryo-Keikaku) and Secondary Medical Areas (Nijiiryoken): The Eighth Medical Care Plan (2024–2029).

[12] Hayashida K., Murakami G., Matsuda S., Fushimi K. (2021). History and profile of Diagnosis Procedure Combination (DPC): Development of a real data collection system for acute inpatient care in Japan. J. Epidemiol..

[13] Reich M.R., Ikegami N., Shibuya K., Takemi K. (2011). 50 years of pursuing a healthy society in Japan. Lancet.

[14] Wennberg J., Gittelsohn A. (1973). Small area variations in health care delivery. Science.

[15] Diehr P., Cain K., Connell F., Volinn E. (1990). What is too much variation? The null hypothesis in small-area analysis. Health Serv. Res..

[16] Corallo A.N., Croxford R., Goodman D.C., Bryan E.L., Srivastava D., Stukel T.A. (2014). A systematic review of medical practice variation in OECD countries. Health Policy.

[17] Brownlee S., Chalkidou K., Doust J., Elshaug A.G., Glasziou P., Heath I., Nagpal S., Saini V., Srivastava D., Chalmers K. (2017). Evidence for overuse of medical services around the world. Lancet.

[18] Berwick D.M., Hackbarth A.D. (2012). Eliminating waste in US health care. JAMA.

[19] Organisation for Economic Co-operation and Development (2023). Integrated care for people living with multiple chronic conditions: A policy brief. Health at a Glance 2023.

[20] Shinjo D., Aramaki T. (2012). Geographic distribution of healthcare resources, healthcare service provision, and patient flow in Japan: A cross sectional study. Soc. Sci. Med..

[21] Sun Y., Sakata N., Iwagami M., Yoshie S., Inokuchi R., Ito T., Kuroda N., Hamano J., Tamiya N. (2024). Regional disparities in home health care utilization for older adults and their associated factors at the secondary medical area level: A nationwide study in Japan. Geriatr. Gerontol. Int..

[22] Kusunoki T., Yoshikawa T. (2024). The distribution structure of medical and care resources based on regional characteristics throughout Japan in 2020. BMC Health Serv. Res..

[23] Seo Y., Takikawa T. (2022). Regional variation in national healthcare expenditure and health system performance in central cities and suburbs in Japan. Healthcare.

[24] Hosokawa R., Ojima T., Myojin T., Aida J., Kondo K., Kondo N. (2020). Associations between healthcare resources and healthy life expectancy: A descriptive study across secondary medical areas in Japan. Int. J. Environ. Res. Public Health.

[25] Hosokawa R., Ojima T., Myojin T., Aida J., Kondo K., Kondo N. (2023). Association between the standardized mortality ratio and healthy life expectancy in Japan. JMA J..

[26] Fukuda Y., Nakamura K., Takano T. (2005). Cause-specific mortality differences across socioeconomic position of municipalities in Japan, 1973–1977 and 1993–1998: Increased importance of injury and suicide in inequality for ages under 75. Int. J. Epidemiol..

[27] Fukuda Y., Nakamura K., Takano T. (2007). Higher mortality in areas of lower socioeconomic position measured by a single index of deprivation in Japan. Public Health.

[28] Fujita M., Sato Y., Nagashima K., Takahashi S., Hata A. (2016). Income related inequality of health care access in Japan: A retrospective cohort study. PLoS ONE.

[29] Halloran M.E., Rothman K.J., Greenland S., Lash T.L. (2008). Ecological measures of effect and ecological studies. Modern Epidemiology.

[30] Andersen R.M. (1995). Revisiting the Behavioral Model and access to medical care: Does it matter?. J. Health Soc. Behav..

[31] Babitsch B., Gohl D., von Lengerke T. (2012). Re-revisiting Andersen’s Behavioral Model of Health Services Use: A systematic review of studies from 1998–2011. Psychosoc. Med..

[32] Xin Y., Ren X. (2023). Determinants of province-based health service utilization according to Andersen’s Behavioral Model: A population-based spatial panel modeling study. BMC Public Health.

[33] von Elm E., Altman D.G., Egger M., Pocock S.J., Gøtzsche P.C., Vandenbroucke J.P. (2007). The Strengthening the Reporting of Observational Studies in Epidemiology (STROBE) statement: Guidelines for reporting observational studies. Lancet.

[34] Statistics Bureau of Japan 2020 Population Census of Japan. https://www.e-stat.go.jp/.

[35] Ministry of Health, Labour and Welfare (Japan) Survey of Physicians, Dentists and Pharmacists; Survey of Medical Institutions; and Hospital Report. https://www.e-stat.go.jp/.

[36] Ministry of Health, Labour and Welfare (Japan) Municipal-Level Vital Statistics in Japan, 2020–2024 (Standardized Against the 2015 National Reference). https://www.e-stat.go.jp/.

[37] Geospatial Information Authority of Japan Digital National Land Information (Administrative-Area Data and Secondary Medical Area Shapefile). https://nlftp.mlit.go.jp/ksj/.

[38] Aichi Prefectural Government Welfare Administrative Releases 2024: Welfare-Benefit Recipient Rate and Long-Term-Care Certification Rate. https://www.pref.aichi.jp/.

[39] Zeileis A. (2006). Object-oriented computation of sandwich estimators. J. Stat. Softw..

[40] Bates D., Mächler M., Bolker B., Walker S. (2015). Fitting linear mixed-effects models using lme4. J. Stat. Softw..

[41] Kuznetsova A., Brockhoff P.B., Christensen R.H.B. (2017). lmerTest package: Tests in linear mixed effects models. J. Stat. Softw..

[42] Benjamini Y., Hochberg Y. (1995). Controlling the false discovery rate: A practical and powerful approach to multiple testing. J. R. Stat. Soc. Ser. B Stat. Methodol..

[43] Tamiya N., Noguchi H., Nishi A., Reich M.R., Ikegami N., Hashimoto H., Shibuya K., Kawachi I., Campbell J.C. (2011). Population ageing and wellbeing: Lessons from Japan’s long-term care insurance policy. Lancet.

[44] Hamada S., Takahashi H., Sakata N., Jeon B., Mori T., Iijima K., Yoshie S., Ishizaki T., Tamiya N. (2019). Household income relationship with health services utilization and healthcare expenditures in people aged 75 years or older in Japan: A population-based study using medical and long-term care insurance claims data. J. Epidemiol..

[45] Iihara K., Nishimura K., Kada A., Nakagawara J., Ogasawara K., Ono J., Shiokawa Y., Aruga T., Miyachi S., Nagata I. (2014). Effects of comprehensive stroke care capabilities on in-hospital mortality of patients with ischemic and hemorrhagic stroke: J-ASPECT study. PLoS ONE.

[46] Lee S. (2022). Spatial and socioeconomic inequalities in accessibility to healthcare services in South Korea. Healthcare.

[47] Yamasaki L., Sakamoto H., Hashimoto H., Kondo N., Kunii O., Tsugawa Y., Abe S.K., Ezoe S., Egawa S., Guinto R.R. (2026). Japan’s health system toward 2040: Structural challenges and a renewed social contract. Lancet Reg. Health West. Pac..

