# Supplementary material for: Area-Level Correlates of Inpatient Care Self-Sufficiency in Subnational Health Planning Areas: A Disease-Stratified Ecological Study from Aichi Prefecture, Japan
Source: Healthcare (Basel). 2026 Oct 6;14(19):3322. doi: 10.3390/healthcare14193322 (PMC13665486; doi:10.3390/healthcare14193322)
Supplement: Supplementary file 1 [file healthcare-14-03322-s001.zip › healthcare-4541924-supplementary.pdf]

Supplementary Table S1

Characteristics of the 11 secondary medical areas in Aichi Prefecture (region-by-region values with summary statistics).

Characteristic	Nagoya/ Owari-C	Ama	Owari-E	Owari-W	Owari-N	Chita	Mikawa-NW	Mikawa-SW	Mikawa-SE	East-Mikawa-N	East-Mikawa-S	Median (IQR)	Range
Demographic and geographic													
Area, km²	368	208	230	193	296	392	951	364	444	1,052	1,159	364 (230–392)	193–1,159
Population density, per km²	6,790.1	1,560.0	2,067.5	2,665.1	2,484.4	1,603.1	509.5	1,928.8	962.1	50.1	94.2	1,603.1 (962.1–2,484.4)	50.1–6,790.1
Aged 0–14 years, %	12.05	12.43	14.17	12.84	13.01	13.79	13.52	14.16	14.34	10.25	11.06	13.01 (12.43–14.16)	10.25–14.34
Aged ≥65 years, %	25.25	27.68	24.86	27.64	26.51	25.16	22.68	22.25	23.41	38.60	31.21	25.25 (23.41–27.68)	22.25–38.60
Aged ≥75 years, %	13.32	14.45	12.88	14.46	13.72	12.93	10.70	11.02	11.23	20.70	16.06	13.32 (12.88–14.45)	10.70–20.70
Healthcare supply: facilities													
Hospitals, count	114	11	18	18	26	19	20	22	15	3	10	18 (12.5–20)	3–114
Hospitals per km²	0.309	0.053	0.078	0.093	0.088	0.048	0.021	0.060	0.034	0.003	0.009	0.053 (0.034–0.088)	0.003–0.309
Hospitals per 100,000 pop.	4.56	3.38	3.78	3.50	3.54	3.02	4.13	3.14	3.51	5.69	5.07	3.54 (3.38–4.13)	3.02–5.69
General clinics, count	2,082	193	319	310	412	345	235	348	226	32	104	319 (216.5–348)	32–2,082
General clinics per km²	5.65	0.93	1.39	1.60	1.39	0.88	0.25	0.96	0.51	0.03	0.09	0.93 (0.51–1.39)	0.03–5.65
Healthcare supply: beds													
General beds per km²	41.98	5.39	15.21	14.98	11.38	6.42	2.28	8.48	3.72	0.19	0.62	5.39 (3.72–14.98)	0.19–41.98
Psychiatric beds per km²	11.20	2.30	5.41	4.47	3.65	2.19	0.74	1.08	1.57	0.00	0.20	2.19 (1.08–3.65)	0.00–11.20
Healthcare supply: workforce													
Physicians per km²	18.09	2.67	8.35	5.77	4.40	2.54	0.90	3.16	1.70	0.06	0.19	2.67 (1.70–5.77)	0.06–18.09
Physicians per 100,000 pop.	266.4	171.0	403.9	216.6	177.2	158.2	175.9	163.8	176.5	115.6	172.9	176.5 (171.0–216.6)	115.6–403.9
Healthcare need (SMR, 2020–2024)													
All-cause SMR	102.2	101.6	88.0	96.5	93.6	96.4	88.5	99.5	90.9	101.7	96.8	96.5 (90.9–101.6)	88.0–102.2
All-cancer SMR	95.3	97.3	84.5	88.8	86.7	90.0	82.3	88.7	84.3	78.8	85.3	86.7 (84.3–90.0)	78.8–97.3
Cerebrovascular SMR	68.5	70.1	63.3	67.8	72.4	68.5	63.7	76.5	78.2	128.6	94.2	70.1 (67.8–78.2)	63.3–128.6
Cerebral infarction SMR	59.7	64.7	60.1	58.6	63.3	62.0	61.5	70.1	77.7	146.9	98.1	63.3 (60.1–77.7)	58.6–146.9
Liver disease SMR	99.7	91.7	83.8	95.3	80.9	91.1	91.2	94.9	69.9	75.8	83.4	91.1 (83.4–94.9)	69.9–99.7
COPD SMR	76.4	73.3	58.2	75.7	58.3	65.5	52.2	70.0	64.9	94.8	78.6	70.0 (64.9–76.4)	52.2–94.8

Characteristic	Nagoya/ Owari-C	Ama	Owari-E	Owari-W	Owari-N	Chita	Mikawa-NW	Mikawa-SW	Mikawa-SE	East-Mikawa-N	East-Mikawa-S	Median (IQR)	Range
Socioeconomic (SES)													
Welfare benefit, per 1,000 population	19.01	7.27	3.47	8.41	7.69	4.79	5.05	4.23	6.22	4.34	6.49	6.22 (4.56–7.48)	3.47–19.01
Elderly single HH, %	34.2	23.1	25.0	23.6	25.6	24.6	20.2	22.2	22.2	22.4	23.2	23.2 (22.3–24.8)	20.2–34.2
Elderly couple HH, %	28.2	28.9	34.3	29.2	32.8	31.2	30.9	27.1	30.4	28.8	26.5	29.2 (28.5–31.0)	26.5–34.3
≥1 elderly HH, %	33.6	44.3	37.2	43.7	39.7	38.4	33.8	34.8	36.3	61.3	42.3	38.4 (35.5–43.0)	33.6–61.3
LTC certification, %	20.1	16.9	16.5	16.8	16.3	16.9	15.3	15.5	16.2	18.3	15.2	16.5 (15.8–16.9)	15.2–20.1

Values are shown for each of the 11 secondary medical areas (SMAs) of Aichi Prefecture, together with the median (interquartile range) and range across the 11 SMAs. Demographic and household-composition data are from the 2020 Population Census of Japan. Physician counts are from the 2022 Survey of Physicians, Dentists and Pharmacists; hospital, clinic, and bed counts are from the FY2024 Survey of Medical Institutions and the Hospital Report; the welfare-benefit recipient rate and the long-term-care certification rate are from 2024 Aichi prefectural administrative releases. Density indicators (per km²) use the total area of each SMA derived from the Digital National Land Information. Standardized mortality ratios (SMRs) were computed by indirect standardization using the 2015 national age- and sex-specific cause-specific death rates as the reference, with deaths pooled over 2020–2024 (5-year pool); values above 100 indicate higher-than-expected mortality. The six cause-specific SMRs shown are the pre-specified primary inferential set (Table 2 of the main text); all 18 cause-specific SMRs are reported in Table 3 of the main text. HH, household; IQR, interquartile range; LTC, long-term care; SMA, secondary medical area; SMR, standardized mortality ratio.

Supplementary Table S2. Definitions, units, reference years, and data sources of all outcome and explanatory variables.

Block	Variable	Definition	Unit	Reference year/period	Data source
Outcome	Care self-sufficiency rate (primary outcome)	Admissions of residents of SMA i treated in hospitals located in SMA i, divided by all admissions of residents of SMA i, computed for each MDC × surgical-stratum cell (resident-based denominator)	%	FY2024	DPC Form-1 (203 hospitals)
Outcome	Outflow rate	100 − self-sufficiency rate (resident-based complement)	%	FY2024	Derived from DPC Form-1
Outcome	Inflow rate	Admissions to hospitals located in SMA i contributed by residents of other SMAs, divided by all admissions to hospitals in SMA i (provider-based denominator)	%	FY2024	DPC Form-1
Outcome	Net Inflow Index (NII)	Inflow rate minus outflow rate	Percentage points	FY2024	Derived from DPC Form-1
Demographic	Aged ≥75 years	Population aged ≥75 years as a share of total population	%	2020	Population Census of Japan
Supply	Physician density	Physicians working at medical institutions per km² of SMA area	per km²	2022	Survey of Physicians, Dentists and Pharmacists (MHLW)
Supply	Hospital density	Hospitals per km² of SMA area	per km²	FY2024	Survey of Medical Institutions (MHLW)
Supply	General-bed density	General hospital beds per km² of SMA area	per km²	FY2024	Survey of Medical Institutions / Hospital Report (MHLW)
Supply	Psychiatric-bed density	Psychiatric hospital beds per km² of SMA area	per km²	FY2024	Survey of Medical Institutions / Hospital Report (MHLW)
Need	Cause-specific SMRs (6 pre-specified; 18 in the exploratory screen)	Indirect standardization using the 2015 national age- and sex-specific cause-specific death rates as the reference; observed deaths pooled over 2020–2024; 100 = parity with the national reference	Ratio (×100)	2020–2024 (2015 reference)	Vital Statistics in Japan (MHLW)
Socioeconomic	Welfare-benefit recipient rate	Public-assistance recipients per 1,000 population	per 1,000	2024	Aichi prefectural administrative releases
Socioeconomic	Elderly single-person household rate	One-person private households in which the person is aged ≥65 years, as a share of all private households	%	2020	Population Census of Japan
Socioeconomic	Elderly couple-only household rate	Couple-only private households with at least one member aged ≥65 years, as a share of all private households	%	2020	Population Census of Japan
Socioeconomic	Households with ≥1 elderly member	Private households including at least one member aged ≥65 years, as a share of all private households	%	2020	Population Census of Japan
Socioeconomic	Long-term-care certification rate	Residents certified as requiring long-term care or support as a share of the population aged ≥65 years	%	2024	Aichi prefectural administrative releases
Geographic	SMA area and Rook adjacency	Total area of each SMA (denominator of the density indicators) and first-order Rook contiguity between SMAs (spatial weights for Model B and Moran's I)	km² / binary	—	Digital National Land Information (GSI)

SMA, secondary medical area; MDC, Major Diagnostic Category; DPC, Diagnosis Procedure Combination; SMR, standardized mortality ratio; NII, Net Inflow Index; MHLW, Ministry of Health, Labour and Welfare; GSI, Geospatial Information Authority of Japan; FY, fiscal year. All explanatory variables were aggregated to the SMA level and z-standardized before model fitting. FY2024 denotes the Japanese fiscal year 2024 (1 April 2024 to 31 March 2025); the SMR period 2020–2024 denotes calendar years of death with 2015 national reference rates. The reference year/period column gives the time period covered by each data source.
